# Global research trends and emerging focus areas in IgG4-related disease: a bibliometric and visual analysis

**DOI:** 10.1186/s41065-025-00489-2

**Published:** 2025-07-01

**Authors:** Ye Li, Zhen-Yu Huang, Ya-Yu Li

**Affiliations:** 1 Department of Nephrology, Hangzhou TCM Hospital of Zhejiang Chinese Medical University, Hangzhou, 310000 Zhejiang Province China; 2 Key Laboratory of Kidney Disease Prevention and Control Technology, Department of Nephrology, Hangzhou Hospital of Traditional Chinese Medicine, No. 453 of Tiyuchang Road, Xihu District, Hangzhou, 310012 Zhejiang Province China

**Keywords:** Bibliometrics, Cite space, IgG4-RD, Research hotspots, Visual analysis, VOS viewer

## Abstract

**Background:**

Immunoglobulin G4-related disease (IgG4-RD) is a systemic autoimmune condition marked by fibroinflammatory infiltration, which may present as solitary or multiple masses and organ enlargement. In advanced stages, progression to multi-organ dysfunction or failure may occur, potentially resulting in mortality. This study aimed to examine the current status and evolving research trends in IgG4-RD from 2020 to 2024 through bibliometric and visualization methodologies.

**Method:**

A total of 1,084 English-language publications on IgG4-RD, dated from January 1, 2020, to October 22, 2024, were retrieved from the Web of Science Core Collection. Bibliometric and visual analyses were performed using VOSviewer and CiteSpace software to identify research trends, collaborative networks, and thematic developments.

**Results:**

The analysis included 1,084 publications, reflecting sustained research activity in the field over the examined period. The Chinese Academy of Medical Sciences & Peking Union Medical College accounted for the greatest number of publications. Among individual contributors, Wen Zhang was identified as the most prolific author. In terms of national output, China led in publication volume, whereas Japan exhibited the highest total citation frequency. *Internal Medicine* published the greatest number of articles, while *Modern Rheumatology* had the highest citation rate. Keyword co-occurrence analysis revealed prominent research themes, including “IgG4-related disease,” “autoimmune pancreatitis,” “case report,” “Rituximab,” and “Diagnosis.”

**Conclusion:**

The global research output on IgG4-RD has demonstrated notable growth in recent years. Further investigations focusing on the emerging thematic areas may yield valuable insights that can enhance clinical diagnosis and therapeutic approaches for individuals affected by IgG4-RD.

## Introduction

Immunoglobulin G4-related disease (IgG4-RD) is a systemic autoimmune disorder involving multiple organ systems. The condition most frequently affects the lacrimal glands, salivary glands, pancreas, kidneys, lymph nodes, aorta, and retroperitoneum. Its characteristic pathological feature is fibroinflammatory infiltration, which may manifest as solitary or multiple masses and organ enlargement, potentially progressing to organ dysfunction, failure, or mortality [1]. Clinical presentations vary considerably and largely depend on the organs involved. Frequently reported symptoms include weight loss, jaundice, abdominal pain, cough, hemoptysis, dyspnea, and lower extremity edema [2, 3, 4]. 

The pathogenesis of IgG4-RD remains incompletely elucidated. Current evidence implicates both B cells and T cells involvement; however, their precise mechanisms require further clarification [2]. Current investigations primarily focus on genetic predispositions, environmental triggers, and immunological dysregulation [5]. Epidemiological data on the incidence and prevalence of IgG4-RD remain limited. In Asia, the estimated annual incidence ranges from 0.28 to 1.08 per 100,000 individuals. In Japan, annual case estimates range from 336 to 1,300. The condition predominantly affects middle-aged and older males, with reported male-to-female ratios ranging from 1.6:1 to 4:1 [5, 6]. 

Early identification and treatment are critical in delaying fibrotic progression and minimizing irreversible organ damage. In 2019, the American College of Rheumatology (ACR) and the European League Against Rheumatism (EULAR) collaboratively developed international classification criteria for IgG4-RD. These criteria provide a standardized diagnostic framework that incorporates clinical, serological, radiological, and pathological features [7]. Glucocorticoids (GCs) currently constitute the first-line therapeutic approach and are generally associated with rapid symptom resolution. In addition, immunosuppressive agents and biologic therapies have demonstrated favorable efficacy in selected clinical scenarios [5, 8, 9]. 

The present bibliometric analysis aimed to examine the global research activity on IgG4-RD during the period from January 1, 2020, to October 22, 2024. Using VOSviewer and CiteSpace software, the study sought to map prevailing international research themes and emerging developmental trends to support future investigations and inform clinical practice.

## Materials and methods

### Literature search

The publications included in this study were retrieved from the Web of Science Core Collection (WOSCC), a widely recognized and authoritative database frequently utilized for bibliometric research and indexing over 10,000 peer-reviewed journals [10]. The literature search was conducted on October 22, 2024, targeting studies published between January 1, 2020, and October 22, 2024. The following search terms were applied: “immunoglobulin G4-related disease,” “IgG4-RD,” and “IgG4 related disease.” The search was confined to the Topic field, which comprises titles, abstracts, author keywords, and author information. Inclusion criteria were restricted to original research articles and review papers. Publications such as early online releases, conference abstracts, and other non-research literature were excluded. Only English-language documents were considered. Following the application of these criteria, a total of 1,084 publications were identified and included in the final analysis.

### Data extraction and analysis

VOSviewer (version 1.6.20.0) and CiteSpace (version 6.3 R3) were employed to conduct bibliometric and visualization analyses. These tools facilitated the exploration of collaborative relationships among countries or regions, institutions, journals, and authors. In the generated network maps, nodes and connecting lines represented clusters and their relationships, with larger nodes indicating higher publication volume or keyword frequency. Shorter or thicker lines denoted closer collaborative ties. Keyword co-occurrence analysis was conducted with a time overlay to illustrate shifts in research focus. In this overlay, earlier research themes were represented by purple nodes, while more recent or emerging topics appeared in yellow. CiteSpace was also employed to identify burst keywords—terms that experienced a marked increase in citation frequency over a short time—highlighting emerging areas of academic interest. CiteSpace facilitated keyword clustering, which grouped thematically similar terms to highlight predominant research themes. These clusters were then chronologically arranged based on the initial year of keyword appearance, resulting in a cluster timeline view. This hybrid representation, combining cluster analysis with temporal mapping, enabled detailed assessment of the development and evolution of specific thematic areas in the field of IgG4-RD.

## Results

### Annual publication trends

**Fig. 1:**
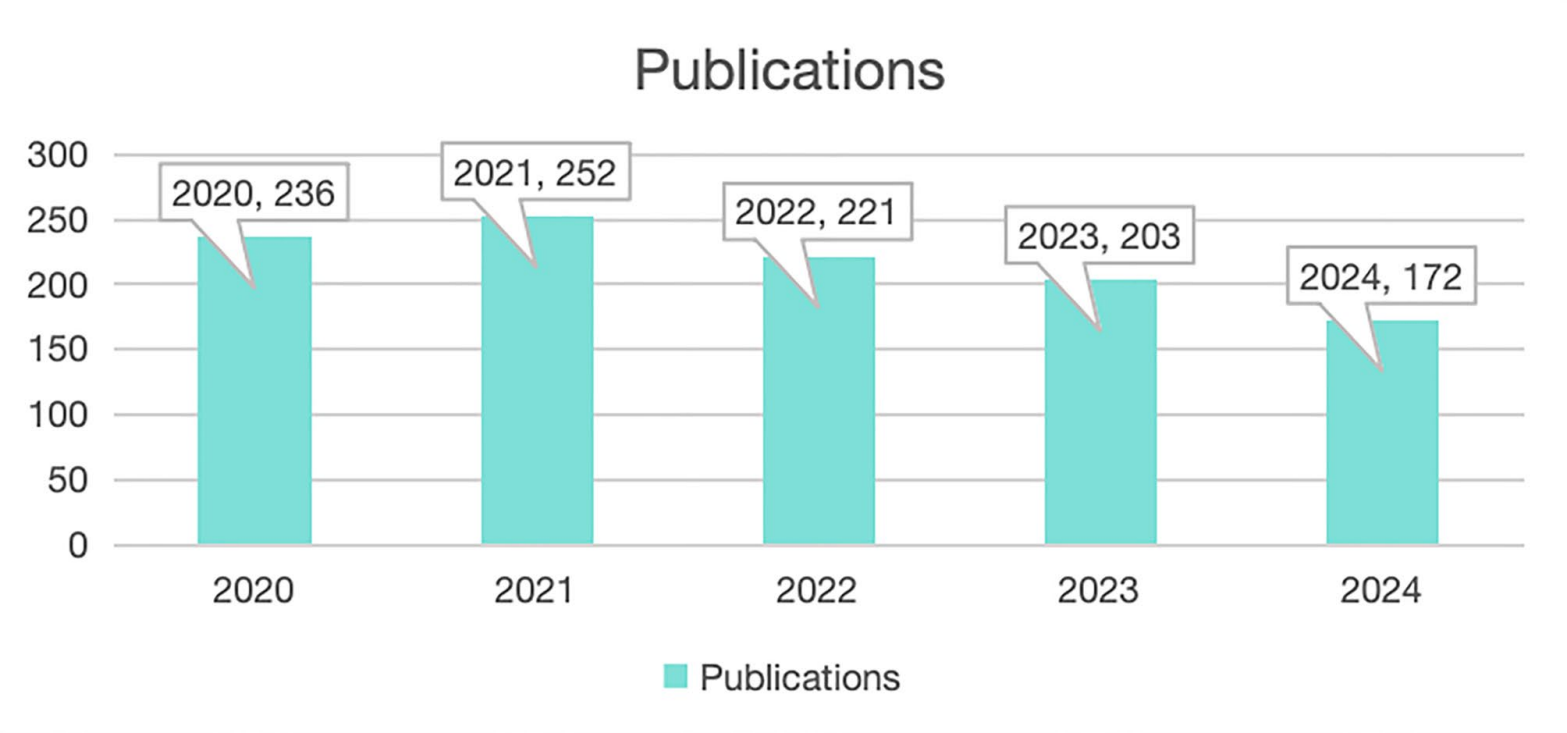
Annual publication trends related to IgG4-RD in the WOSCC (2020–2024)

A total of 1,084 publications related to IgG4-RD, dated between January 1, 2020, and October 22, 2024, were retrieved from the WOSCC. As illustrated in Fig. 1, the annual volume of publications remained consistently above 170, reflecting sustained academic interest and establishing IgG4-RD as a continued research focus. The highest output was observed in 2021, with 252 articles and 980 citations recorded for that year. Across all included publications, the total citation count reached 7,087, corresponding to an average of 6.54 citations per article. The H-index for the overall academic output on IgG4-RD during this period was calculated as 31, indicating both productivity and citation impact.

### Authors of publications

**Fig. 2:**
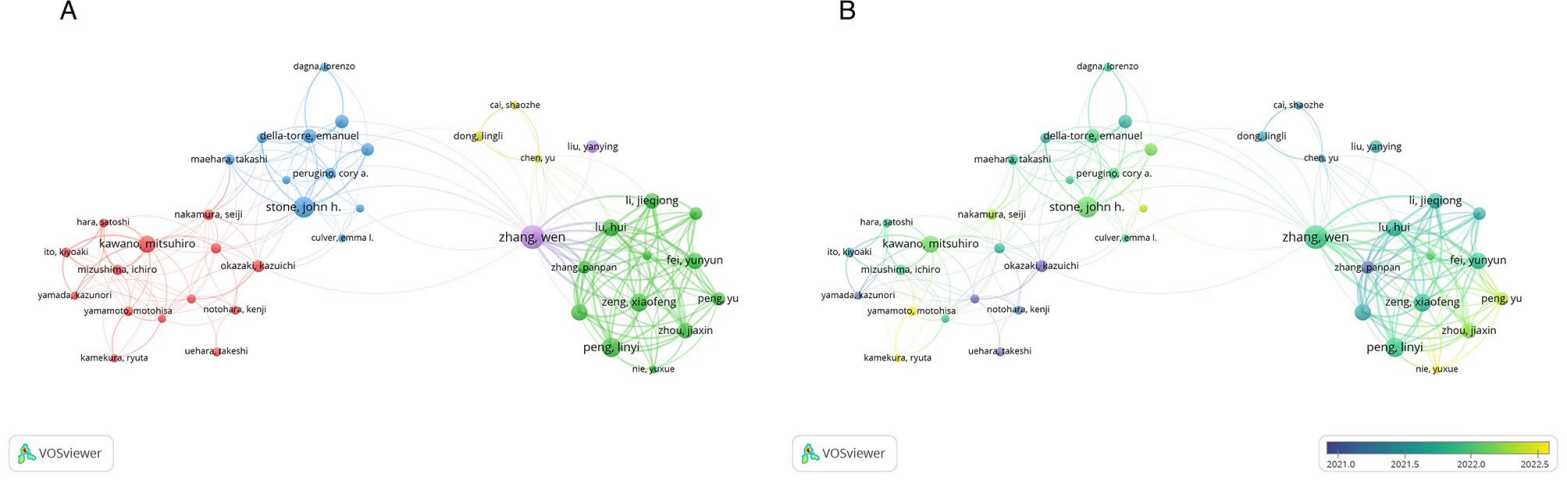
Author collaboration networks and temporal distribution in IgG4-RD research (WOSCC, 2020–2024). (**A**) Co-authorship network of authors with ≥ 9 publications. (**B**) Temporal overlay visualization illustrating the evolution of author contributions, with purple nodes indicating early contributors and yellow nodes representing more recent authors

A total of 5,945 individual authors contributed to the 1,084 publications analyzed from the WOSCC. Table 1 presents the five most prolific authors, with Wen Zhang contributing the highest number of publications (*n* = 49), followed by John H. Stone (*n* = 38). Author collaboration networks were visualized using VOSviewer, applying a threshold of at least nine publications per author, which resulted in the inclusion of 42 authors. The network map (Fig. 2A) classified these authors into five clusters, with the largest (red cluster) comprising 14 authors. An overlay visualization using a timeline approach (Fig. 2B) indicated that Kazuichi Okazaki and Shigeyuki Kawa were among the earliest contributors to IgG4-RD research during the examined period. Wen Zhang emerged as a prominent author during the mid-phase of the study period, while Motohisa Yamamoto and Ryuta Kamekura were identified as recent contributors, indicating emerging influence in this field.

**Table 1:** Top five authors by publication volume in IgG4-RD research (WOSCC database, 2020–2024)

Author	Number of publications	Total citations	Average citations per article
Wen Zhang	49	546	11.14
Stone, John H.	38	1135	29.87
Peng, Linyi	31	346	11.16
Zeng, Xiaofeng	29	242	8.34
Della-Torre, Emanuel	28	854	30.5

### Journals of publication

The 1,084 publications on IgG4-RD were distributed across 418 journals. Table 2 presents the ten journals with the highest number of publications. Among them, *Internal Medicine* published the most articles (44 articles), followed by *Frontiers in Immunology* (41 articles) and *Rheumatology* (36 articles). Citation analysis indicated that *Modern Rheumatology* had the highest average citation count per article among the top-ranked journals, with 22 articles receiving an average of 17.09 citations each. *Rheumatology* followed closely in citation performance. These findings indicate that both journals maintain significant influence in the field of autoimmune disease research. Additionally, among the top ten journals, *Frontiers in Immunology* had the highest impact factor, indicating strong academic visibility and disciplinary relevance.

**Table 2:** Ten journals with the highest publication count in IgG4-RD research (WOSCC database, 2020–2024)

Rank	Journal title	Number of publications	Total citations	Average citations per article
1	INTERNAL MEDICINE	44	91	2.07
2	FRONTIERS IN IMMUNOLOGY	41	259	6.32
3	RHEUMATOLOGY	36	471	13.08
4	MEDICINE	26	49	1.88
5	MODERN RHEUMATOLOGY	22	376	17.09
6	CLINICAL RHEUMATOLOGY	21	96	4.57
7	ARTHRITIS RESEARCH THERAPY	19	187	9.84
8	JOURNAL OF CLINICAL MEDICINE	17	126	7.41
9	CLINICAL AND EXPERIMENTAL RHEUMATOLOGY	16	65	4.06
10	RHEUMATOLOGY INTERNATIONAL	15	70	4.67

### Countries and regions of publication

**Fig. 3:**
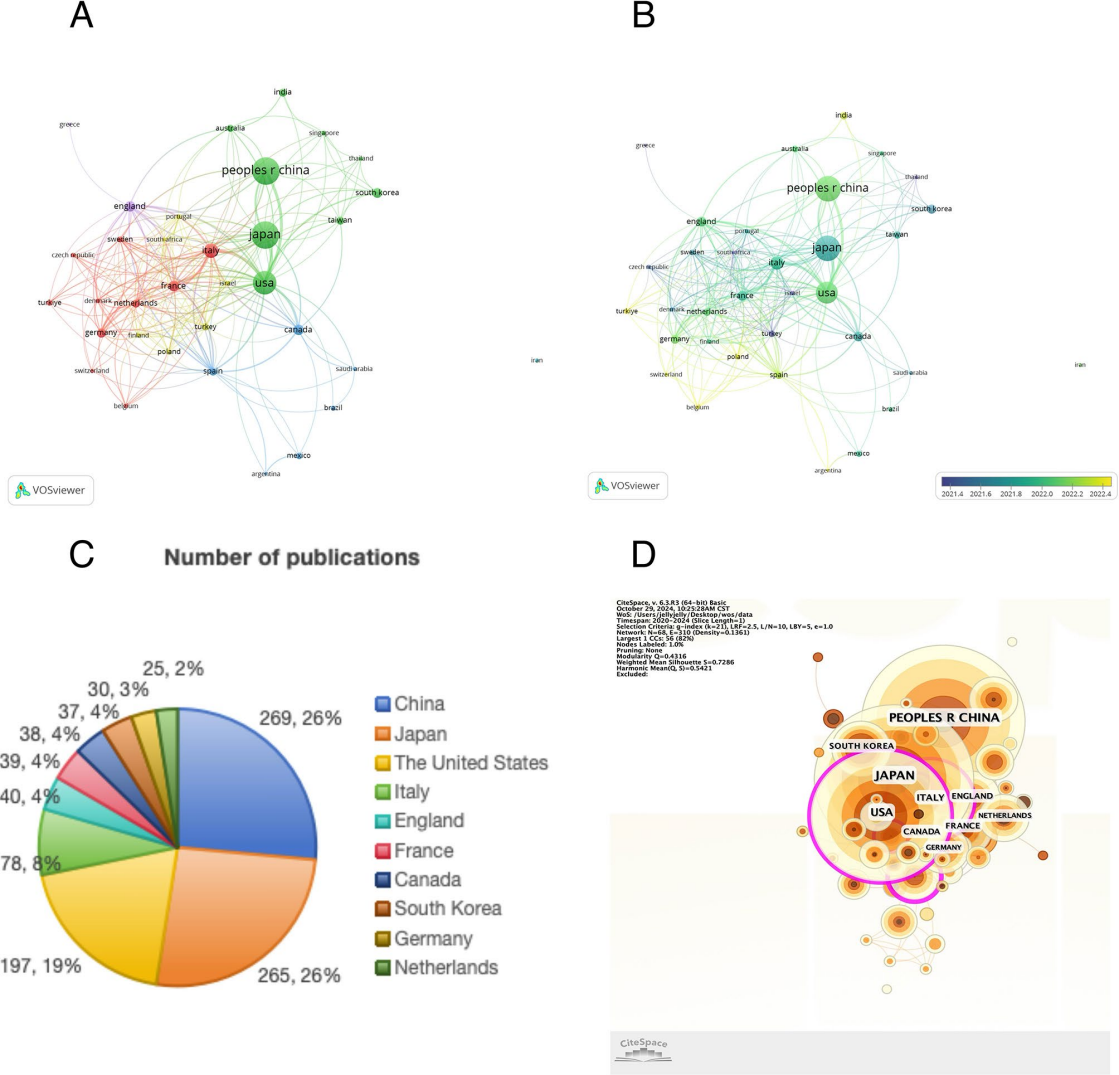
Geographic distribution and collaboration in IgG4-RD research (WOSCC, 2020–2024). (**A**) Co-authorship network visualization of countries/regions with ≥ 5 publications. (**B**) Temporal overlay map showing the evolution of country-level contributions; earlier contributors (e.g., Japan, Italy) presented in purple and more recent contributors (e.g., Poland, Turkey) in yellow. (**C**) Sector diagram presenting the top 10 countries/regions by publication volume on IgG4-RD in the WOSCC database (2020–2024). (**D**) Co-occurrence map illustrating international collaboration patterns

The 1,084 articles retrieved from the WOSCC originated from 68 countries and regions. As presented in Table 3, the 10 most productive countries in terms of publication volume were China, Japan, the United States, Italy, England, France, Canada, South Korea, Germany, and the Netherlands. China ranked first with 269 publications, followed by Japan and the United States. (Fig. 3C) Japan recorded the highest total citation count, while Italy recorded the highest average citations per article. These findings indicate that both countries have made influential contributions to IgG4-RD research. Although the United States and Japan produced a higher volume of publications and total citations, average citation counts per article were lower than those of Italy. In contrast, South Korea exhibited relatively low total and average citation metrics, indicating potential for further growth in this research area.

**Table 3:** Top ten countries/regions by publication output in IgG4-RD research (WOSCC database, 2020–2024)

Country	Number of publications	Publication rank	Total citations	Total citations rank	Average citations per article	Average citations per article rank	Total collaboration strength	Collaboration strength rank
China	269	1	1388	4	5.16	9	51	6
Japan	265	2	1982	1	7.48	8	86	3
USA	197	3	1836	2	9.32	6	159	1
Italy	78	4	1494	3	19.15	1	106	2
England	40	5	359	9	8.98	7	65	4
France	39	6	508	5	13.03	4	63	5
Canada	38	7	457	6	12.03	5	46	9
South Korea	37	8	137	10	3.7	10	12	10
Germany	30	9	417	7	13.9	3	47	8
Netherlands	25	10	394	8	15.76	2	50	7

Collaborative relationships among countries and regions were visualized using VOSviewer (Fig. 3A), with inclusion limited to those contributing at least five publications. Among the 34 countries/regions meeting this criterion, the United States demonstrated the greatest total link strength (159), followed by Italy (106), Japan (86), England (65), and France (63). Regarding publication chronology, China and the United States began contributing slightly later than Japan and Italy, whereas countries such as Poland and Turkey have emerged more recently within the field. (Fig. 3B) CiteSpace analysis (Fig. 3D) demonstrated inter-country collaboration networks, highlighting particularly strong academic ties between the United States and countries including Japan and Italy.

### Contributing institutions

**Fig. 4:**
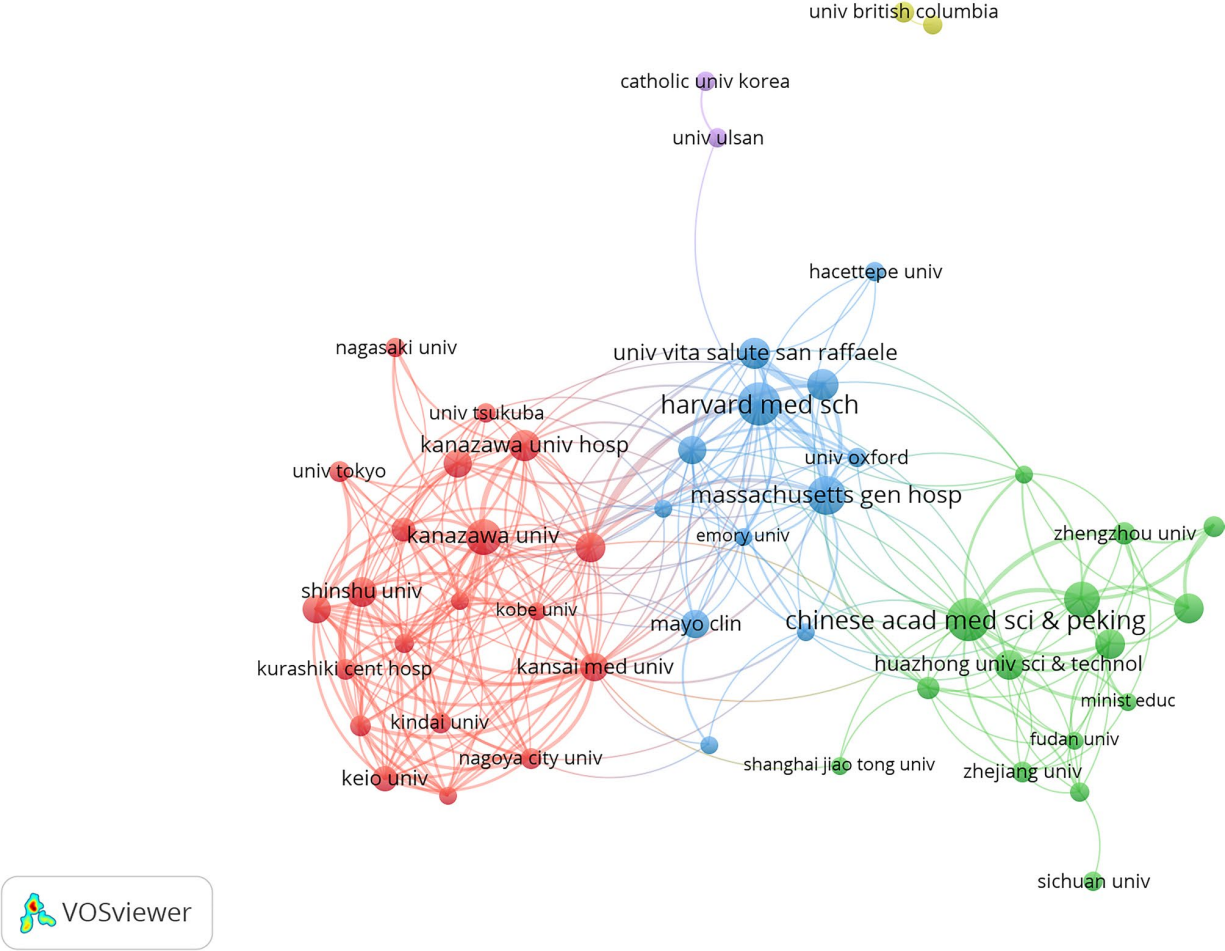
Institutional contributions and collaboration networks in IgG4-RD research in the WOSCC database

A total of 1,542 institutions were identified as contributors to the 1,084 articles analyzed. Table 4 presents the ten institutions with the highest publication output. The Chinese Academy of Medical Sciences & Peking Union Medical College ranked first, followed by Harvard University and Peking Union Medical College Hospital. Among these top institutions, four are based in China, three in the United States, two in Italy, and one in Japan, demonstrating China’s leading position in terms of publication volume on IgG4-RD. Institutional collaboration networks were further examined using VOSviewer, with a minimum publication threshold of eight articles. Among the 51 institutions meeting this inclusion criterion, Kanazawa University, Harvard University, and Harvard Medical School demonstrated the highest total link strengths, as presented in Fig. 4.

**Table 4:** Ten institutions with the highest publication output in IgG4-RD research (WOSCC database, 2020–2024)

Institution	Number of publications	Total citations	Average citations per article	Total collaboration strength	Collaboration strength rank
CHINESE ACADEMY OF MEDICAL SCIENCES PEKING UNION MEDICAL COLLEGE	62	485	7.82	45	6
HARVARD UNIVERSITY	59	1275	21.61	66	2
PEKING UNION MEDICAL COLLEGE HOSPITAL	54	435	8.06	13	10
PEKING UNION MEDICAL COLLEGE	51	306	6	14	9
MASSACHUSETTS GENERAL HOSPITAL	49	1258	25.67	63	4
HARVARD MEDICAL SCHOOL	48	1161	24.19	66	3
KANAZAWA UNIVERSITY	41	633	15.44	82	1
VITA SALUTE SAN RAFFAELE UNIVERSITY	33	910	27.58	46	5
IRCCS OSPEDALE SAN RAFFAELE	33	910	27.58	45	7
CAPITAL MEDICAL UNIVERSITY	29	80	2.76	29	8

### Analysis of the most frequently cited publications

An analysis of the most frequently cited articles offers insights into the global research priorities and influential contributions within the field of IgG4-RD. Table 5 presents the ten most cited publications, along with their respective citation counts and thematic areas. These highly referenced studies represent foundational or influential work that has shaped ongoing research and may serve as valuable references for investigators in defining future study directions.

**Table 5:** Ten most frequently cited articles in IgG4-RD research (WOSCC database, 2020–2024)

Rank	Title	Citation count	Research content
1	The 2020 revised comprehensive diagnostic (RCD) criteria for IgG4-RD	258	Revised Comprehensive Diagnostic (RCD) Criteria for IgG4-RD developed in 2020
2	Hashimoto’s thyroiditis: An update on pathogenic mechanisms, diagnostic protocols, therapeutic strategies, and potential malignant transformation	234	Advances in the study of IgG4-RD—Hashimoto’s thyroiditis: covering pathogenesis, diagnostic basis, and treatment strategies
3	IgG4-related disease: an update on pathophysiology and implications for clinical care	191	The role of B cells and CD4+ T cells in the pathogenesis of IgG4-RD and its clinical implications
4	Advances in the diagnosis and management of IgG4 related disease	177	Advances in the pathophysiology, diagnosis, and personalized treatment strategies for IgG4-RD
5	Rosai-Dorfman disease: an overview	133	Clinical correlation between Rosai-Dorfman disease and IgG4-RD
6	Disentangling inflammatory from fibrotic disease activity by fibroblast activation protein imaging	124	Differentiating inflammatory and fibrotic activity in IgG4-RD: Implications for targeted anti-fibrotic therapies
7	The 2019 American College of Rheumatology/European League Against Rheumatism classification criteria for IgG4-related disease	123	2019 ACR and EULAR collaboration on the development of new international classification criteria for IgG4-RD
8	European Guideline on IgG4-related digestive disease - UEG and SGF evidence-based recommendations	118	Diagnostic and management guidelines for IgG4-related gastrointestinal diseases in European adults and children
9	Fibroblast Activation Protein-Targeted PET/CT with 68Ga-FAPI for Imaging IgG4-Related Disease: Comparison to 18 F-FDG PET/CT	109	Ga-68-FAPI as a potential imaging agent for assessing IgG4-RD
10	IgG4-related disease: a clinical perspective	86	Recent clinical advances in IgG4-RD: A clinical perspective

### Keywords

The 10 most frequently occurring keywords are presented in Table 6, with “autoimmune pancreatitis,” “case report,” “Rituximab,” and “Diagnosis” appearing most frequently. A total of 1,084 publications related to IgG4-RD, retrieved from the WOSCC, were analyzed using VOSviewer for keyword mapping and clustering. This analysis provided insights into the current research status and prominent thematic areas within the IgG4-RD field. From the dataset, 2,154 keywords were identified using VOSviewer, of which 133 occurred 5 times or more. After consolidating redundant terms, such as variations of “IgG4” (e.g., “IgG4-RD” and “Immunoglobulin G4”), 125 keywords were retained for co-occurrence and cluster analysis.

**Table 6:** Ten most frequently occurring keywords in IgG4-RD research (WOSCC database, 2020–2024)

Keywords	Frequency	Frequency ranking
IgG4-related disease	492	1
autoimmune pancreatitis	75	2
case report	52	3
Rituximab	42	4
Diagnosis	30	5
retroperitoneal fibrosis	29	6
Treatment	26	7
Inflammation	24	8
autoimmune diseases	22	9
Relapse	20	10

**Fig. 5:**
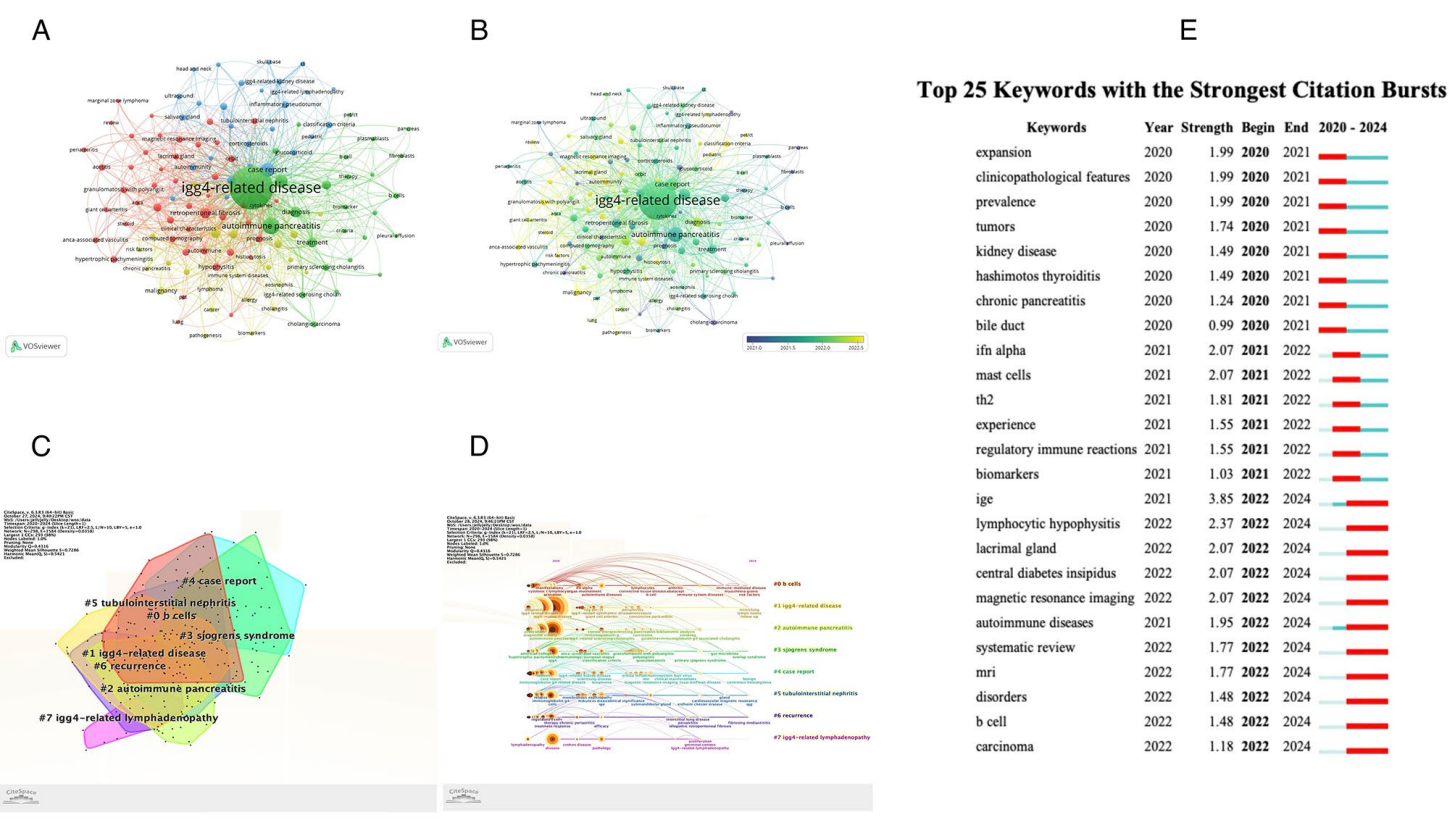
Keyword analysis and thematic evolution in IgG4-RD research in the WOSCC database. (**A**) Network visualization of keyword co-occurrence. (**B**) Time overlay visualization highlighting early and emerging research hotspots. (**C**) Keyword clustering visualization. (**D**) Keyword clustering timeline. (**E**) Top 25 emerging keywords ranked by burst intensity

As presented in Fig. 5A, keyword clustering delineated four major thematic areas: ① Cluster 1 encompassed IgG4-RD manifestations across various organs and tissues, including terms such as aortitis, giant cell arteritis, hypophysitis, membranous nephritis, retroperitoneal fibrosis, and tubulointerstitial nephritis. ② Cluster 2 focused on the pathogenesis, diagnostic standards, and treatment approaches associated with IgG4-RD. Keywords in this cluster included diagnosis, biomarker, B cell, fibrosis, plasmablast, PET/CT, criteria, therapy, and Rituximab. ③ Cluster 3 was characterized by clinical variants and case-based studies, incorporating terms such as case report, IgG4-related kidney disease, IgG4-related lymphadenopathy, IgG4-related ophthalmic disease, IgG4-related tubulointerstitial nephritis, prognosis, and pediatric. ④ Cluster 4 addressed autoimmune conditions associated with IgG4-RD, including autoimmune diseases, Sjögren’s syndrome, systemic lupus erythematosus, rheumatoid arthritis, and cancer.

Temporal overlay mapping (Fig. 5B) revealed evolving research priorities. Purple nodes indicate earlier hotspots, whereas yellow nodes highlight more recent areas of interest. Between 2020 and 2022, keywords such as “relapse,” “lymphadenopathy,” “prognosis,” “pancreatitis,” and “pleural effusion” were predominant. In contrast, the period from 2023 to 2024 saw increased emphasis on terms such as “pediatric,” “classification criteria,” “malignancy,” “pathogenesis,” and “autoimmune diseases,” indicating a shift in research priorities within the field.

Through an analysis conducted using CiteSpace software, the keywords were categorized into 8 distinct clusters, each exhibiting a clustering module value (Q value) greater than 0.3, signifying a reliable clustering structure, and an average silhouette value (S value) exceeding 0.7, confirming strong and consistent clustering structures. The extracted cluster label keywords are presented in Fig. 5C: #0 B cells, #1 IgG4-related disease, #2 autoimmune pancreatitis, #3 Sjogren’s syndrome, #4 case report, #5 tubulointerstitial nephritis, #6 recurrence, and #7 IgG4-related lymphadenopathy.

The addition of a temporal dimension to the clusters, as depicted in Fig. 5D, reveals a shift in research focus over time. Initial emphasis was placed on terms such as “manifestations,” “diagnosis,” “relapse,” “treatment response,” and “lymphadenopathy”, while mor recent attention has shifted toward “IgG,” “risk factors,” “lymph nodes,” “gut microbiota,” and “cavernous hemangioma.” Moreover, the top 25 emerging keywords are presented in Fig. 5E, with high-intensity keywords (≥ 2) including “magnetic resonance imaging,” “central diabetes insipidus,” “lacrimal gland,” “lymphocytic hypophysitis,” “IgE,” “mast cells,” and “IFN alpha.” Additionally, it is evident that earlier research primarily concentrated on “kidney disease,” “clinicopathological features,” and “prevalence,” while more recent studies have increasingly focused on topics such as “carcinoma,” “B cells,” “MRI,” and “IgE.”

## Discussion

This bibliometric analysis examined global research trends related to IgG4-RD over the past five years, comprising a total of 1,084 articles selected from the WOSCC. Data processing was performed using VOS viewer software, enabling co-occurrence analysis across multiple parameters, including authors, countries/regions, institutions, citation counts, and keywords. This approach provides an insightful overview of the current research status and emerging trends in the field.

### Current research status of IgG4-RD

Over the past five years, the volume of publications on IgG4-RD has consistently exceeded 170 manuscripts annually, indicating sustained global interest in the field. This ongoing attention may be attributed to the 2019 international classification criteria for IgG4-RD, which were endorsed by the ACR and EULAR [7]. These authoritative diagnostic criteria have provided a robust framework for clinical diagnosis, thus facilitating further exploration and research into the disease. Notably, the year 2021 marked the peak in research output, with 252 publications, likely due to the release of revised diagnostic criteria and guidelines for IgG4-related kidney disease and gastrointestinal diseases in multiple regions [11, 12]. The high number of clinical case report studies published in 2021 further highlights the growing interest among clinicians in IgG4-RD.

Analysis of WOSCC data identified Wen Zhang of the Department of Rheumatology at Peking Union Medical College Hospital, Chinese Academy of Medical Sciences, as the most prolific author in the field, contributing 49 publications between 2020 and October 2024, accumulating 546 citations, with an average of 11.14 citations per article. His research primarily focuses on the clinical features and therapeutic outcomes of IgG4-RD across various systems, including urological diseases, ophthalmic disorders, aortitis/adventitial inflammation, and pericarditis, as well as thyroiditis [13, 14, 15, 16]. In a retrospective study on IgG4-related urological disease, Zhang et al. observed that patients typically responded well to initial immunosuppressive therapy, although relapse was noted during the maintenance phase of the treatment [13]. John H. Stone of Harvard Medical School ranked as the second most prolific author, contributing 38 publications with a total of 1,135 citations, with an average of approximately 29.87 citations per article. This citation impact underscores the influence and recognition of his work. Stone’s research has emphasized the immunopathogenesis of IgG4-RD, including the development of new international classification criteria and the evaluation of treatment efficacy [7, 17, 18]. His research has notably focused on the role of B cells in tissue fibrosis in patients with IgG4-RD, and the mechanisms through which CD4 + and CD8 + cytotoxic T lymphocytes may induce mesenchymal stem cell apoptosis, thereby contributing to disease progression [17, 19, 20]. These studies have significantly advanced the clinical understanding of IgG4-RD pathophysiology.

Among the ten journals with the highest publication volumes on IgG4-RD, *Frontiers in Immunology* holds the highest impact factor. The journal focuses on the clinical and immunological phenotyping of human immune diseases and aims to define their molecular mechanisms, with a strong emphasis on cutting-edge progress in immunology. In contrast, *Modern Rheumatology* is the most highly cited journal in the field of IgG4-RD and is dedicated to publishing research in rheumatology and related disciplines.

From a geographical perspective, China, Japan, the United States, and Italy are the top four countries in terms of publication volume on IgG4-RD, with their articles also ranking among the most highly cited. These countries are key contributors to current research on the disease. Japan ranks first in total citation count, with 1,982 citations. In 2020, Japanese researchers published a revised comprehensive diagnostic (RCD) criterion for IgG4-RD, which built upon the 2010 diagnostic standards by introducing new concepts in clinical and radiological features, serological diagnosis, and pathological diagnosis, thereby enhancing diagnostic sensitivity and specificity. This revision has been widely recognized by researchers globally [21]. 

The United States, Italy, and Japan also rank among the top three countries for overall collaborative strength, reflecting extensive inter-country research networks. This collaboration has contributed to the development of new ideas and advancements in research. While China ranks first in publication volume, its total citation count and overall collaboration strength are relatively lower, indicating a need for improving publication quality and strengthening international collaboration to achieve more impactful research outcomes. Among the top ten most productive institutions, China holds a leading position in terms of output volume. However, in terms of collaborative strength, institutions in Japan, the U.S., and Italy outperform those in China, which aligns with the earlier findings.

### Research trends in IgG4-RD

Bibliometric analysis indicates that the ten most frequently cited articles primarily address the pathogenesis, diagnosis, and recent advancements in the treatment of IgG4-RD. These studies have significantly contributed to the establishment of new authoritative diagnostic and classification criteria for the disease. In the European context, new guidelines have been introduced for the diagnosis and management of IgG4-related diseases affecting the digestive system. These efforts aim to enhance diagnostic accuracy, promote effective treatment strategies, and improve prognostic outcomes.

Among the ten most frequently occurring keywords, “Rituximab,” “Treatment,” and “Relapse” were prominent, indicating substantial scholarly interest in the therapeutic application of Rituximab in IgG4-RD, as well as increasing attention to disease relapse and prognosis. The principal therapeutic objectives in IgG4-RD management include inflammation control, alleviation of symptoms, preservation of organ function, and minimization of adverse effects. GCs remain the foundational treatment modality. An initial prednisone dosage of 30 to 40 mg/day is generally considered appropriate, while long-term maintenance therapy with prednisone at doses ≥ 5 mg/day may contribute to relapse prevention [22]. Although combined therapy involving GCs and immunosuppressive agents has been shown to reduce serum IgG4 concentrations, its efficacy and safety profile may be suboptimal in certain individuals. Consequently, Rituximab, an anti-CD20 monoclonal antibody, has garnered considerable attention in recent research. Administration of Rituximab frequently results in a significant reduction in serum IgG4 levels. As a B cell-depleting agent, Rituximab has demonstrated efficacy not only in symptom management but also in maintenance therapy, contributing to relapse reduction [23, 24, 25]. In individuals with renal impairment, Rituximab has been shown to lower IgG4 levels, improve glomerular filtration rate, and enhance clinical and histopathological outcomes [25]. The keyword, “case report” also emerged as a frequently cited term and formed a prominent cluster in the VOSviewer analysis. Case reports play a critical role in elucidating complex clinical presentations, underlying mechanisms, diagnostic approaches, and therapeutic interventions. In the context of IgG4-RD, they are frequently employed to document rare manifestations involving atypical organs or tissues, such as the lumbar spine, oral cavity, heart, peripheral nerves, and gastrointestinal tract [26, 27, 28, 29, 30]. 

Cluster analysis conducted using VOSviewer identified current research hotspots within the field of IgG4-RD, with the most prominent cluster centered on organ- and tissue-specific manifestations of the disease. Notably, retroperitoneal fibrosis (RPF) emerged as one of the ten most frequently cited keywords. Idiopathic retroperitoneal fibrosis (IRPF) accounts for approximately two-thirds of RPF cases and is recognized as a chronic, nonspecific inflammatory condition of the retroperitoneal tissue characterized by fibrosclerotic proliferation. IRPF typically affects the abdominal aorta and the iliac arteries distal to the renal arteries. Elevated serum IgG4 levels, together with dense infiltration of IgG4-positive plasma cells observed on tissue biopsy, support the classification of a subset of IRPF cases as IgG4-related RPF. Clinically, affected individuals often present with nonspecific symptoms such as fever, anorexia, hydronephrosis, abdominal or lumbar pain, and peripheral edema. Involvement of the urinary tract frequently results in obstructive uropathy [31, 32]. The primary therapeutic objective in such cases is the alleviation of ureteral obstruction. Surgical interventions, including nephrostomy and ureteral stent placement, are commonly employed as first-line treatments. Pharmacological management may involve glucocorticoids, immunosuppressive agents, and biologics [33, 34]. 

Another prominent research within the IgG4-RD domain is autoimmune pancreatitis (AIP), a manifestation of IgG4-RD that affects the pancreas. Type 1 AIP is characterized by elevated serum IgG4 concentrations and increased infiltration of IgG4-positive plasma cells in pancreatic tissue. In contrast, type 2 AIP is not associated with IgG4. The absence of definitive biomarkers continues to complicate the differentiation between autoimmune pancreatitis and malignancies, particularly pancreatic cancer [35]. Bibliometric analysis further indicated that “malignancy” emerged as a global research hotspot in IgG4-RD literature during 2023–2024. Epidemiological studies conducted in China reported an increased incidence of malignancy among individuals with IgG4-RD, with a positive association observed between autoimmune pancreatitis and cancer development [36]. Additionally, mild to moderate elevations in CA 19 − 9 levels have been observed in individuals with autoimmune pancreatitis [37]. Japanese researcher Minaga K. identified a strong association between serum IFN-α, IL-33, and IgG4 concentrations, suggesting their potential utility as novel biomarkers for distinguishing IgG4-RD and autoimmune pancreatitis from pancreatic cancer [38]. Further investigations by Minaga K. indicated that overactivation of plasmacytoid dendritic cells (pDCs) leads to excessive production of type I interferons (IFN-I) and IL-33, which contribute to persistent inflammation and fibrosis, thereby playing a role in autoimmune pathogenesis. These findings suggest that targeting the pDC pDC–IFN-I–IL-33 axis may represent a promising therapeutic approach. Agents such as anti-ST2 antibodies (anti-ST2 Ab) and Etokimab— which inhibit IL-33 signaling, are currently under investigation for their potential to mitigate inflammation and fibrosis in individuals with AIP and IgG4-RD [39]. 

In recent years, the research focus in the field of IgG4-RD has evolved. Analysis of keyword bursts identified “B cell” and “IgE” as emerging areas of emphasis, suggesting increasing scholarly interest in elucidating the immunopathogenic mechanisms underlying the disease. Additionally, Temporal overlay visualizations further indicate that “pathogenesis” is likely to remain a key area of interest in the future, offering potential avenues for the development of novel therapeutic strategies. Clinically, IgG4-RD can present with elevated serum IgE levels, with studies demonstrating a significant correlation between IgE and IgG4 concentrations. Higher serum IgE levels are often associated with increased disease activity in IgG4-RD [40]. Studies have demonstrated that IgG4-IgE immune complexes can be detected in patients with IgG4-RD, indicating that these complexes may play a role in the pathogenesis of the disease. Allergic symptoms are commonly observed in patients with IgG4-RD. Allergic symptoms are frequently observed in individuals with IgG4-RD, and repeated exposure to certain allergens can stimulate IL-4 production by Th2 cells and IL-10 secretion by Treg cells, both of which contribute to IgG4 class switching. Furthermore, elevated IgE levels and predisposition to allergic responses have been identified as risk factors for IgG4-RD relapse [41, 42, 43]. 

The significant clinical improvement observed in patients with IgG4-RD following B cell depletion with Rituximab further underscores the role of B cells in the disease’s pathogenesis. Further evidence has been provided by Takashi Maehara et al. who demonstrated that IgG4-secreting plasma cells and activated B cells form part of an inflammatory network through interaction with disease-specific CD4⁺ T cells. This interaction is implicated in organ damage and tissue fibrosis [44, 45]. In tissue samples from individuals with IgG4-related retroperitoneal fibrosis (IgG4-RPF), an increased proportion of B cells has been observed, with a notable rise in activated B cell subsets. These activated B cells exhibit elevated expression of costimulatory molecules, including CD70 and ADGRE5 (CD97), and are actively involved in cytokine secretion linked to immune activation and fibrotic responses, further implicating them in disease pathogenesis [46]. Furthermore, keywords such as “pediatric,” “classification criteria,” and “malignancy” have also demonstrated upward trends in recent analyses, potentially leading to valuable research findings.

## Limitations

This study is subject to several limitations. Although the Web of Science is regarded as one of the most authoritative literature search tools, it does not encompass all the literature in the IgG4-RD field. Publications indexed in non-Science Citation Index (SCI) journals and other databases were not included. Additionally, this study focused solely on articles published in English, thereby omitting potentially valuable studies published in other languages, a limitation that has been acknowledged in prior bibliometric studies [47]. The search was further constrained to publications containing the terms “immunoglobulin G4-related disease,” “IgG4-RD,” or “IgG4 related disease” in the title, abstract, or keywords, excluding literature where these terms appeared in the main body of the text, which may have contributed to an incomplete dataset.

## Conclusion

This bibliometric study analyzed global research trends and thematic developments in the field of IgG4-RD between 2020 and 2024, using software-based analysis to identify research hotspots while also predicting future research directions. These findings provide valuable a comprehensive overview of the evolving research landscape and provide strategic guidance for future investigations. Ongoing research are expected to focus on the elucidating the pathogenesis of IgG4-RD, refining and standardizing the international classification criteria, enhancing diagnostic accuracy, and advancing more effective treatments. Additionally, increasing attention is anticipated in the diagnosis and management of IgG4-RD in pediatric populations.

## Data Availability

All data generated or analysed during this study are included in this article. Further enquiries can be directed to the corresponding author.

## Abbreviations

IgG4-RD: IgG4-related disease
ACR: American college of rheumatology
EULAR: European league against rheumatism
GCs: Glucocorticoids
WoSCC: Web of science core collection
RCD: Revised comprehensive diagnostic
AIP: Autoimmune pancreatitis
IFN-α: Interferon-alpha
IL-33: Interleukin-33
IgG4: Immunoglobulin G4
pDCs: Plasmacytoid dendritic cells
IFN-I: Type I interferon
IL-4: Interleukin-4
Th2: T helper 2
IL-10: Interleukin-10
T cell: Treg cell
